# Policy of King Edward's Hospital Fund

**Published:** 1921-02-05

**Authors:** 


### THE PRESERVATION OF THE VOLUNTARY SYSTEM.

### Policy of King Edward's Hospital Fund.

### A MEMORABLE OCCASION.

A special meeting of the President and General Council of King Edward's Hospital Fund for London was held at St. James's Palace on January 26 to consider the policy to be recommended to the Government for the preservation of the voluntary system of hospital management and control.

In the unavoidable absence of H.R.H. the Prince of Wales, President of the Fund, the Right Hon. James W. Lowther, M.P. (Speaker of the House of Commons), presided. Those present were: The Bishop of Southwark, Cardinal Bourne, Rev. A. E. Garvie, D.D., Rev. W. T. A. Barber, D.D., the Earl of Iveagh, the Viscount Mersey, the Viscount Burnham, the Viscount Finlay, Lady Ampthill, Lord Stamfordham, Lord Somerleyton (Hon. Secretary), Lord Stuart of Wortley, Lord Marshall, the Hon. Oswald Partington, the Chairman of the County Council (Mr. John W. Gilbert), the President of the Royal College of Surgeons (Sir Anthony A. Bowlby), Sir Francis H. Champneys, Bart., Lieut.-General Sir Alfred Keogh, Sir Edward Hope, Major-General Sir George Makins, Sir John Rose Bradford, Sir William H. Bennett, Sir J. Kingston Fowler, Sir William J. Collins, Sir Frederick M. Fry (Hon. Secretary), Sir Alan G. Anderson (Hon. Secretary), Sir Frederick Green, Sir George Lawson Johnston, Sir Cooper Perry, Sir John Craggs, Sir John Tweedy, Mr. John G. Griffiths, Mr. Leonard L. Cohen, Professor Winifred Cullis, Mr. Yvon R. Eccles, Dr. H. Morley Fletcher, Mr. Henry L. Hopkinson, Mr. Kenneth Prescott.

Sir Alan Anderson (Hon. Secretary) read an order, signed by H.R.H. the President, appointing Mr. Fred Hall, M.P., a member of the Council, in the place of Mr. Will Crooks, M.P., resigned.

Lord Stuart of Wortley (the Chairman of the Executive Committee) presented a report of the Committee on the subject of the policy to be recommended to the Government for the preservation of the voluntary system of hospital management and control, in accordance with the resolution of the General Council of December 14 last. The specific resolutions contained therein [see below], having been seconded by Lord Burnham, and supported by Sir William Collins and Sir John Craggs, were unanimously adopted and ordered to be forwarded to the Prime Minister, the Minister of Health, and the members of the Government Committee of Inquiry.

#### Special Committee Appointed.

The Speaker of the House of Commons announced that a special Committee had been constituted by H.R.H. the President for the purpose of preparing statements within the limits of the general principles approved by the President and General Council, and that His Royal Highness had appointed the following persons as members thereof: Lord Stuart of Wortley (Chairman), Sir Cooper Perry, the Viscount Burnham, Lord Somerleyton, Sir Frederick Fry, and Sir Alan Anderson.

The Council thereupon delegated the necessary powers to the Special Committee.

Cardinal Bourne, in moving a vote of thanks to the Speaker for presiding (which was seconded by Viscount Finlay), said that the present meeting constituted a memorable occasion in the history of the Fund. It seemed to him that their very hearty thanks were due to the Executive Committee who had prepared the recommendations, in that they had succeeded, as far as he could judge, in safeguarding as far as possible the voluntary principle of dealing with the hospitals. They were passing through very difficult and critical times, and in the midst of those difficulties the Committee seemed to him to have shown very great skill.

